# Diet in Pregnancy
*A paper read before the Bristol Medico-Chirurgical Society on Wednesday, April 12th, 1939.


**Published:** 1939

**Authors:** J. A. Nixon

**Affiliations:** Emeritus Professor Medicine in the University of Bristol; Consulting Physician to the Bristol Royal Infirmary, and Southmead Hospital, etc.


### The Bristol Medico-Chirurgical Journal

*"Scire est nescire, nisi id me*
*Scire alius sciret."*

AUTUMN, 1939.

### DIET IN PREGNANCY.*

BY

J. A. Nixon, C.M.G., M.D., F.R.C.P.,

*Emeritus Professor of Medicine in the University of Bristol;*
*Consulting Physician to the Bristol Royal Infirmary,*
*and Southmead Hospital, etc.*

New physiological facts about food travel slowly and long delay is apt to occur before they are applied practically. The outstanding instance is the length of time which elapsed between Lancaster's [1] demonstration of the value of oranges and lemons, and the general adoption of their use in the prevention and cure of scurvy. It is hard to realize that Lancaster persuaded the East India Company to issue these fruits or their juices as a routine item of victualling on all their ships by the year 1600!

The time has come for inquiring whether the present-day dieting of pregnant women is in accordance with

* A paper read before the Bristol Medico-Chirurgical Society on Wednesday, April 12th, 1939.

N

present-day knowledge. There is a mass of evidence showing that pregnant women in Great Britain suffer, at all income levels, from dietary deficiencies.

### CALORIES.

The average calorie-requirement of an active non-pregnant woman amounts to 2,500 calories daily. During the first four months of pregnancy it is probable that no increase is called for. In the later months there may be a greater expenditure of energy, particularly if the woman continues to perform active housework. The calorie-intake should be gradually increased after the fourth month, until at the end of the ninth month the total calories should be about 20 per cent. higher than at the beginning of pregnancy. McCollum [2] has drawn attention to some pertinent observations on the metabolic rate during pregnancy. In the first few months the basal metabolic rate falls very slightly, until in the fifteenth week it is about 1.5 per cent. below the Harris-Benedict standard for a woman of the same height and weight. Then the rate steadily rises until a few days before delivery, when it is about 23 per cent. above that in the fifteenth week. Now a woman's gain in weight during pregnancy is about 15 per cent., which would normally correspond with an increase in metabolism of only 5 per cent. instead of the 23 per cent. observed. It is evident that the fœtal tissues and their adnexa have a higher specific metabolism per unit of weight than that of the unencumbered woman. McCollum goes on to say that " all authorities are agreed that the pregnant woman requires for her nutrition nothing other than those nutrients which she requires in the non-pregnant state, but that she needs more of everything, particularly more energy, protein, calcium, phosphorus, iron and the vitamins." McCollum's "all authorities" may include physiologists, bio-chemists and students of nutrition, but is it true of the medical profession? McCance[3] and his co-workers hold that the advice of present-day obstetricians and gynæcologists (in Great Britain at any rate) is being questioned and challenged by experimental scientists. The scientists demand that a healthy woman should be so fed that she may be able to provide for the requirement of the fœtus and finish her pregnancy without any demand upon her own resources.

The League of Nations Technical Commission[4] has made recommendations which are almost revolutionary in character, especially as regards the optimum protein intake. The following is a "specimen" diet recommended for pregnant and nursing women.

A. PROTECTIVE FOODS.

| | Amount. | | Notes. |
|---|---|---|---|
| | grams. | oz. | |
| Milk .. .. .. .. | 1,000 | 35 | |
| Meat, fish or poultry .. | 120 | 4 | Lean meat. |
| Eggs, one .. .. .. | 50 | 2 | |
| Cheese .. .. .. | 30 | 1 | Cheddar. |
| Green or leafy vegetables .. | 100 | 3½ | |
| Potatoes .. .. .. | 250 | 8 | |
| Legumes, dried .. .. | 10 | ⅓ | Beans. |
| Cod-liver oil .. .. .. | 3·5 | ⅛ | |
| Vitamin C from raw fruit or raw vegetables. | One orange or 1 oz. lemon juice or 2 oz. tomato juice. | — | |

B. SUPPLEMENTARY ENERGY-YIELDING FOODS.

| | Amount. | | Notes. |
|---|---|---|---|
| | grams. | oz. | |
| Cereals: White or whole-meal flour. | 250 | 8 | |

| | Calories. | Protein. grams. | Calcium. grams. | Phosphorus. grams. | Iron. mgm. |
|---|---|---|---|---|---|
| This diet yields .. .. | 3,440 | 105 | 1·6 | 2·0 | 10·2 |
| McCance and his co-workers suggest a lower daily intake except of iron .. | 2,500 | 90 | 1·5 | 2·0 | 20 |

### PROTEIN.

It will be seen that the League of Nations recommendation is an intake of 1 gm. per kilogram during the first three months of pregnancy (the same as for a non-pregnant adult) and 1.5 gm. per kilogram during the last six months. Milhorat,[5] in discussing McCollum's paper, observed that the composition of fœtal proteins is different from that of maternal proteins ; different combinations of the indispensable amino-acids are required. The protein molecule cannot be transported as such from the maternal to the fœtal structures. It must be broken down into the amino-acids and re-synthetized into the fœtal proteins. In order to secure the necessary amounts of the indispensable amino-acids, he suggested that more maternal tissue is broken down than is used in the actual synthesis of the fœtal proteins. McCance *et al.* point out that from the fourth to the ninth month various workers have demonstrated a maternal storage of nitrogen far greater than the needs of the fœtus and adnexa. This nitrogen-retention has been shown to vary directly with the intake. Strauss considered that toxæmias of pregnancy could often be traced to a low protein intake and could be improved by a greatly increased protein intake. Milhorat observed that in bitches fasting during pregnancy led to excessive destruction of maternal proteins with a nitrogen excretion at least 50 per cent. higher per kilogram of body-weight than during the non-pregnant state.

High protein intake cannot be as harmful to healthy woman as some obstetricians think, for the Eskimo woman continues throughout pregnancy to take the high protein diet customary in the country where she lives.

McCance *et al.*, in their studies of diets of pregnant women by the individual method, found that the intake of total protein and of animal protein rose convincingly with income; and it is interesting to note that in each income-group except the lowest the maximum protein intake was considerably higher than 1.5 gm. per kilo. of body-weight, and that the average in each group except the two lowest approximated to the League of Nations allowance of 1.5 gm. There is perhaps a small proportion of pregnant women in whom symptoms of threatened toxæmia warrant a restriction of protein during the later months of pregnancy, but there is no physiological justification for advising as a universal plan diets that are actually deficient in protein. Yet even amongst the laity the fear seems widespread of eating meat, eggs and cheese, or even of drinking milk during pregnancy. Without a study of the chemistry of the blood a general application of low-protein feeding is unnecessary and may be dangerous. If the phosphorus and uric acid content of the blood are above normal, some caution about the protein intake is necessary, but women with a high blood cholesterol, low urea nitrogen and normal blood phosphorus and uric acid are benefited by larger amounts of protein.

### CALCIUM AND PHOSPHORUS.

From the twenty-eighth week of pregnancy up to full term the mother has to supply the fœtus with 25 grams of calcium and 15 grams of phosphorus. The optimum Ca: P ratio for human nutrition has not been ascertained. For rats a ratio of 1 is near the optimum when the calcium level is 0.49 per cent. of the diet. Cox and Imboden,[6] who carried out the experiment on rats, tentatively suggest a calcium requirement for human adults at 1.37 gm. daily.

The League of Nations Technical Commission place it at 1.6 gm., McCance *et al.* recommend 1.5 gm. Norwegian investigations showed that towards the end of pregnancy the calcium need of the mother rises to 1.6 gm., which means about 2½ pints of milk. The phosphorus intake advised by the League of Nations Technical Commission and by McCance *et al.* is 2.0 gm. daily. Phosphorus is present in so many ordinary foods that it is rarely deficient. If the diet of the pregnant woman contains adequate milk and green vegetables she will usually secure sufficient Ca. and P. But in order that these elements shall be properly utilized sufficient Vitamins A and D must be supplied. Another factor which affects calcium and phosphorus metabolism is worry. A deficient supply of calcium will cause a mother to sacrifice that in her own tissues to the needs of the fœtus. When the calcium deficiency is very great osteomalacia may result, and this deficiency may be due to shortage of fats and Vitamin D, not to an absolute lack of calcium. Lesser defects are more common: they are manifested by increase of dental caries during pregnancy, by muscle soreness and weakness which interfere with the performance of ordinary activities and are unrelieved by rest during the day or night. Sometimes severe muscular spasms and cramps make it impossible for the pregnant woman to sit or lie for any length of time in one position.

The best sources of calcium are milk and cheese. Abundant supplies of Vitamin D are required, and it must be remembered that the needs in pregnancy depend in some measure on the amount of sunshine enjoyed. A teaspoonful of cod-liver oil daily (containing about 300 international units of Vitamin D) is recommended by the League of Nations Technical Commission.

### Iron.

Iron is another substance which the mother has to supply in relatively large amounts, and there is ample evidence that deficiency of iron is widespread. Nutritional anæmia is a common disease amongst pregnant and nursing women. It is almost impossible at present to give exact figures for iron requirements during pregnancy. For the adult man Sherman[7] in his latest researches places the daily requirement at 12 mgm., and the needs of the pregnant woman are higher, probably about 20 mgm. But the iron of foods is not all available: for instance, only about 50 per cent. of the iron in wheat, oats and yeast is physiologically available. McCollum points out that ascorbic acid seems to be necessary for iron utilization and that Vitamin C deficiency may be partly responsible for anæmia in pregnancy. At any rate it is fairly definitely proved that when iron is supplied together with abundance of fresh fruit and vegetables anæmic patients respond more readily. Hence it is advisable to give during pregnancy a variety of foodstuffs rich in iron such as meat, egg-yolk, fruit and vegetables.

### Iodine.

Iodine deficiency in the diet of pregnant women may be of great significance. The demands of the fœtus are small but imperative. A certain minimum is necessary to prevent cretinism and goitre, and in some regions it may not be supplied in the food obtainable. Among natural foods sea-fish is the only rich source of iodine and it ought to be included in the diet of all pregnant women once or twice a week. Cod-liver oil is the richest source of iodine in any food, but herrings rank high and are palatable. In districts where endemic goitre is prevalent iodized salt may be used

(1 part of sodium iodide in 200,000 of sodium chloride) bearing in mind that in cases of nodular or adenomatous goitre iodized salt may produce symptoms of thyro-toxicosis.

### Vitamins.

Vitamin deficiencies are common in the diet of pregnant women, particularly deficiency of Vitamin D.

*Vitamin A* deficiency is probably rare in this country: 2,000 to 4,000 international units is a safe daily allowance for an adult and in pregnancy higher amounts may be necessary. It is present in many of the commoner foodstuffs such as cream, butter, liver (especially of fish and sea-birds), animal fat (except as a rule lard), egg-yolk, green leafy vegetables and carrots. Ordinary cooking temperatures scarcely affect Vitamin A.

*Vitamin* $B_1$ may sometimes be deficient in cases of hyperemesis gravidarum, anorexia, and the polyneuritis which occasionally complicates pregnancy.[8] Plass and Mengert[9] have remarked that the recent tendency to force a high carbohydrate diet on patients with vomiting of pregnancy exposes them to a risk of Vitamin $B_1$ deficiency. In cases of persistent vomiting Vitamin $B_1$ can be administered parenterally.

The polyneuritis of pregnancy resembles, both clinically and pathologically, that of alcoholism and beri-beri.

The important part played by Vitamin $B_1$ in carbohydrate metabolism should not be overlooked.

*Vitamin C.*—Reference has already been made to the influence of Vitamin C on iron-assimilation. It also reduces the blood-coagulation time, a matter of great importance to the obstetrician.

*Vitamin D.*—The necessity for adequate supplies of

Vitamin D has been discussed in connection with calcium utilization. Its importance in the prevention of rickets in the offspring and in the production of normal dentition must also be borne in mind.

*Vitamin E* is apparently necessary for the normal development of the fœtus and the completion of pregnancy. Its deficiency is likely to result in abortion, and may be responsible for some cases of habitual abortion. It is present in whole milk, seeds, green leafy vegetables, most vegetable oils (especially wheat-germ oil), whole wheat, egg-yolk, beef and ox liver. Its sources are so numerous and the supply so abundant that deficiencies are probably rare.

*Vitamin K* is known as the antihæmorrhagic factor, and probably acts on the liver, where the reactions essential for the production of pro-thrombin proceed. This vitamin is present in the fat of pigs' liver and also in green vegetables, particularly spinach. Little is known of human deficiencies of Vitamin K.

Many of the foregoing considerations are at present theoretical and require the test of practical application.

### EXPECTANT MOTHERS AND SPECIAL NOURISHMENT.

Some striking facts suggesting that well-fed mothers are less liable to puerperal infection than the under-fed and that they show a much lower death-rate have been brought to light by the nutrition scheme for expectant mothers carried out by the National Birthday Trust Fund (at first) and by the Research Committee of the Joint Council for Midwifery (since April, 1937). Lady Rhys Williams, Honorary Secretary to the Joint Council, has kindly furnished the following table, setting out the results obtained during the period 1st July, 1937, to 31st March, 1939.

PARTICULARS OF THE RESULTS OF THE JOINT COUNCIL OF MIDWIFERY NUTRITIONAL SCHEME FOR EXPECTANT MOTHERS—1st JULY, 1937, TO 31st MARCH, 1939.

| | Total number of mothers delivered. | Number of deaths from sepsis. | Number of deaths from other causes. | Number of deaths from associated causes. | Number of still-births. | Number of neo-natal deaths. | Total infant deaths (still-births plus neo-natal). |
|---|---|---|---|---|---|---|---|
| 1. Cases receiving Special Foods | 15,333 | 4 (0·26) | 36 (2·35) | 17 (1·11) | 528 (34) | 381 (25) | 909 (59) |
| 2. Cases not receiving Special Foods but attending Ante-Natal Clinics .. .. .. .. | 12,010 | 8 (0·67) | 31 (2·58) | 16 (1·33) | 488 (41) | 357 (30) | 845 (71) |
| 3. Cases not receiving Special Foods and not attending Ante-Natal Clinics .. .. .. .. | 21,538 | 19 (0·88) | 77 (3·58) | 31 (1·44) | 1,063 (49) | 919 (43) | 1,982 (92) |
| Total number of cases coming within the scope of the enquiry .. | 48,881 | 31 (0·63) | 144 (2·95) | 64 (1·31) | 2,079 (43) | 1,657 (34) | 3,736 (77) |
| 4. Total cases not receiving Special Foods (*i.e.* (2) and (3) combined) .. .. .. .. | 33,548 | 27 (0·80) | 108 (3·22) | 47 (1·40) | 1,551 (46) | 1,276 (38) | 2,827 (84) |

*Note.*—The data from which the figures have been obtained were kindly supplied by the Medical Officers of Health of the areas concerned. All deaths from abortion are excluded from the above figures. All rates (which are shown in brackets) are calculated per 1,000 total births.

NATIONAL BIRTHDAY TRUST FUND NUTRITION SCHEME FROM 1st JANUARY, 1935, TO 30th JUNE, 1937.

| | Total number of mothers receiving food. | Puerperal death rate from sepsis. | Puerperal death rate from other causes. | Total puerperal death rate. | Maternal death rate from associated causes. | Infant death rate (including still-births). |
|---|---|---|---|---|---|---|
| | | CASES RECEIVING SPECIAL FOOD. | | | | |
| Period 1935–36 .. .. .. | 7,320 | 0·14 | 1·50 | 1·64 | Not available. | Not available. |
| 1937 .. .. .. .. .. | 3,064 | — | 1·63 | 1·63 | 0·98 | 57 |
| Total (1935, 1936 and 1937) .. | 10,384 | 0·09 | 1·54 | 1·63 | — | — |
| | | CASES NOT RECEIVING SPECIAL FOOD. | | | | |
| 1935–36 .. .. .. .. | 14,073 | 3·48 | 2·91 | 6·39 | Not available. | Not available. |
| 1937 .. .. .. .. .. | 4,781 | 1·25 | 4·18 | 5·43 | 2·09 | 102 |
| Total (1935, 1936 and 1937) .. | 18,854 | 2·91 | 3·24 | 6·15 | — | — |

*Note.*—All rates in the above table are calculated per 1,000 total births.

The table of results for the earlier period, published by the National Birthday Trust Fund and quoted in Parliament in 1938,[10] exhibits a similar advantage to the mothers receiving special food, although the table is not constructed in an identical way.

The Research Committee of the Joint Council is very cautious in drawing conclusions from these figures. In the *Interim Report* the possibility of other factors contributing to the apparent superiority of the "fed" mothers is admitted without hesitation. Nevertheless there seem to be some grounds for questioning and challenging the advice of present-day obstetricians and gynæcologists. Further investigations into the true facts are imperatively needed. Is it a fact that the perils of maternity can be lessened by any modifications in the diets of expectant mothers ?

### REFERENCES.

[1] Nixon, J. A., *Proc. Roy. Soc. Med.*, 1937–38, xxxi. 193.

[2] McCollum, E. V., *Amer. Jour. Obst. and Gynec.*, 1938, xxxvi. 586.

[3] McCance, R. A., Widdowson, E. M., and Verdon-Roe, C. M., *Jour. Hyg.*, 1938, xxxviii. 596.

[4] League of Nations. Technical Commission of the Health Committee. *The Problem of Nutrition*, vol ii. "Report on the Physiological Bases of Nutrition." Geneva, 1936.

[5] Milhorat, A. T., *Amer. Jour. Obst. and Gynec.*, 1938, xxxvi. 594.

[6] Cox, W. M., and Imboden, M., *Jour. Nutrition*, 1936, xi. 177.

[7] Sherman, H. C., *Chemistry of Food and Nutrition*, 5th Ed. Macmillan, New York, 1937.

[8] Sinclair, H. M., *Bristol Med.-Chir. Jour.*, 1938, lv. 177.

[9] Plass, E. D., and Mengert, M. D., *J.A.M.A.*, 1933, ci. 2020.

[10] Parliamentary Debates, 17th March and 19th May, 1938, quoted in *Health and Empire*, 1938, xiii. 182.

